# Light pollution of freshwater ecosystems: principles, ecological impacts and remedies

**DOI:** 10.1098/rstb.2022.0360

**Published:** 2023-12-18

**Authors:** Franz Hölker, Andreas Jechow, Sibylle Schroer, Klement Tockner, Mark O. Gessner

**Affiliations:** ^1^ Leibniz Institute of Freshwater Ecology and Inland Fisheries (IGB), 12587 Berlin and 16775 Stechlin, Germany; ^2^ Institute of Biology, Freie Universität Berlin, 14195 Berlin, Germany; ^3^ Senckenberg Society for Nature Research, 60325 Frankfurt Germany; ^4^ Department of BioSciences, Goethe-University, 60438 Frankfurt, Germany; ^5^ Department of Ecology, Berlin Institute of Technology, 10587 Berlin, Germany

**Keywords:** aquatic community dynamics, circadian rhythms, conservation, ecosystem functioning, land–water interactions, light physics

## Abstract

Light pollution caused by artificial light at night (ALAN) is increasingly recognized as a major driver of global environmental change. Since emissions are rapidly growing in an urbanizing world and half of the human population lives close to a freshwater shoreline, rivers and lakes are ever more exposed to light pollution worldwide. However, although light conditions are critical to aquatic species, and freshwaters are biodiversity hotspots and vital to human well-being, only a small fraction of studies conducted on ALAN focus on these ecosystems. The effects of light pollution on freshwaters are broad and concern all levels of biodiversity. Experiments have demonstrated diverse behavioural and physiological responses of species, even at low light levels. Prominent examples are skyglow effects on diel vertical migration of zooplankton and the suppression of melatonin production in fish. However, responses vary widely among taxa, suggesting consequences for species distribution patterns, potential to create novel communities across ecosystem boundaries, and cascading effects on ecosystem functioning. Understanding, predicting and alleviating the ecological impacts of light pollution on freshwaters requires a solid consideration of the physical properties of light propagating in water and a multitude of biological responses. This knowledge is urgently needed to develop innovative lighting concepts, mitigation strategies and specifically targeted measures.

This article is part of the theme issue ‘Light pollution in complex ecological systems’.

## 1. Introduction

Light pollution by artificial light at night (ALAN) is an accelerating environmental problem of global concern. High annual growth rates of 3–6% of night-time sky brightness during the second half of the last century [1] have increased to nearly 10% per year over the last decade [2,3]. This trend is likely to continue as light-emitting diodes (LEDs) make lighting increasingly efficient and affordable [4,5]. The consequent ‘loss of the night’ extends into large areas of unlit regions as light emitted to and scattered in the atmosphere is returned to the Earth. This phenomenon is called skyglow [4] and can today exceed the brightness of full moonlight [6]. Direct light emissions are even several hundred times brighter. The breadth and cross-cutting nature of issues associated with ALAN range from aesthetics to human health and from species to ecosystems [7–9], with the threat to the ecological night niche now being widely recognized, since more than half of the world's described species are nocturnal [10]. In response, great strides have been made in recent years to explore the ecological impacts of artificial light [11–13].

**Figure 1.**
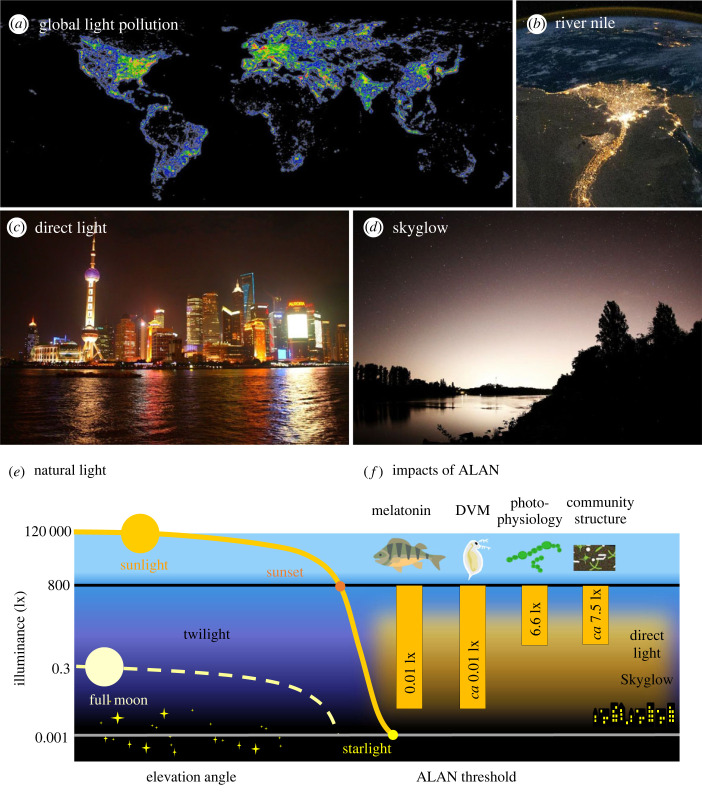
(*a*) Global map of light pollution showing large geographical differences [4]. (*b*) The Nile river and its delta in Egypt as a global hotspot of light pollution (photo: NASA Earth Observatory, 2010). (*c*) Direct light pollution at Huangpu River in Shanghai, China (photo: A. Jechow). (*d*) Indirect light pollution by skyglow over the River Rhine, Germany (photo: A. Jechow). (*e*) Schematic summarizing diel changes in illuminance (note log scale) by major natural light sources during day, twilight and night as a function of elevation angle of Sun and Moon; dark yellow solid line—Sun illuminance on clear day, light yellow dashed line—moonlight full Moon masking natural darkness (adapted from [12]). (*f*) Examples of demonstrated effects [14–17] of low-level light pollution (yellow field) resulting from direct light and skyglow and polluting natural light at night as visualized in (*e*). DVM = diel vertical migration. Fish icon made with Freepik (www.flaticon.com). (Online version in colour.)

Inland waters are under particular pressure, because humans have traditionally settled near water (figure 1) and more than half of the global population today lives within a 3 km perimeter from a freshwater shoreline [18]. Furthermore, despite covering less than 1% of the Earth surface, inland waters host about 10% of all known species and one-third of all vertebrates, more than 125 000 species in total [19]. These facts notwithstanding, ecological impacts of light pollution have mainly been studied in terrestrial [13] and, to some extent, in marine [20] and estuarine [21] ecosystems, whereas inland waters remain greatly understudied [22,23]. Relating insights from terrestrial ecosystems to inland waters is not straightforward, however, as the optical properties of water lead to marked differences in irradiance, spectral composition and polarization patterns in aquatic environments. This implies specific physiological and behavioural adaptations of freshwater organisms, with likely consequences at the population, community and ecosystem levels.

The objective of the present review is to provide a succinct synthesis of current knowledge on light characteristics, light pollution and the ecological consequences of ALAN in inland waters and coupled adjacent ecosystems. The focus is on recent insights into effects at all levels of ecological organization and on ways to alleviate them. Given the importance of optics to understand light regimes and impacts, we begin with a brief characterization of light impinging on water surfaces and propagating under water before addressing the nature, extent and ecological consequences of light pollution. We finish by drawing attention to important gaps in our understanding of light pollution and by highlighting promising lighting concepts, strategies and measures to alleviate the impacts of ALAN on inland waters, which partly differ from principles that have been found or proposed to be effective in other environments.

## 2. Light properties and pollution

### (a) Natural light

Light is defined as that part of the electromagnetic spectrum visible to humans, with wavelengths between 380 and 780 nm, situated between ultraviolet (UV) and infrared (IR) radiation. Although light shares properties of both waves and particles, it is generally sufficient when assessing ecological effects of light pollution to view light as a stream of individual light particles (photons). This includes the physics of light emission, absorption and scattering. Light polarization, however, is best described with the wave nature of light.

**Figure 2.**
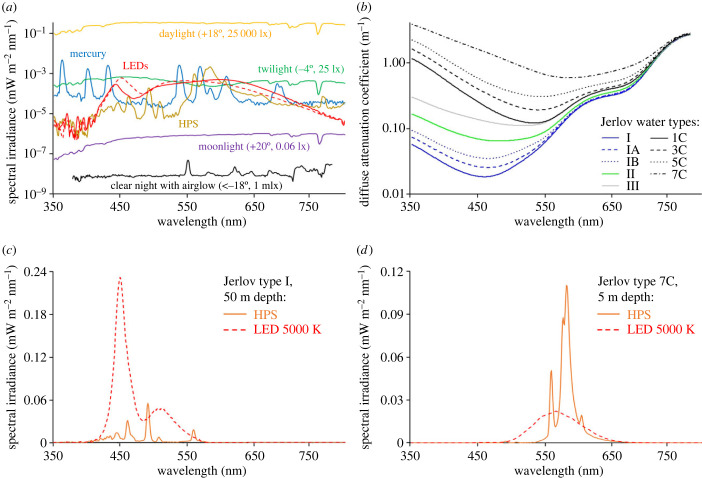
Empirical or modelled data on light spectra in relation to surface waters (A. Jechow 2023, unpublished data). (*a*) Selected spectra (log scale) of natural light sources, including daylight (25 000 lx, Sun 18° above horizon), twilight (25 lx, Sun 4° below horizon), light of a near full Moon (60 mlx, 88% illumination, 20° above horizon), night-time light with airglow (1 mlx, Sun more than 18° below horizon), and various artificial light sources at 25 lx, including high-pressure sodium lamps (HPS), mercury vapour lamps and LEDs at 3000 and 5000 K. (*b*) Spectral diffuse attenuation coefficients (log scale) for different Jerlov water types, covering clear ocean water (type I) to very turbid inland and coastal waters (type 7C) [24]. (*c*) Spectral irradiance of light from HPS lamps and 5000 K LEDs after passing through 50 m of clear ocean water (Jerlov water type I). (*d*) Spectral irradiance of light from HPS lamps and 5000 K LEDs after passing through 5 m of turbid inland and coastal waters (Jerlov water type 7C). (Online version in colour.)

The energy carried by a photon is inversely proportional to the wavelength of light, which many organisms perceive as colour. Natural and artificial light sources usually emit light at many wavelengths, which together constitute the light spectrum (figure 2*a*). Sunlight has a continuous broadband spectrum comprising nearly all wavelengths spanning from UV radiation to the visible range and IR radiation, and the spectrum of moonlight is similar, although usually slightly shifted towards longer (red) wavelengths (figure 2*a*). The exact spectra of sunlight and moonlight vary to some extent, depending for example on altitude [25], and airglow also affects the spectrum of the natural night sky [26].

Natural light incident on the Earth's surface varies by many orders of magnitude between day and night depending on the position of the Sun and the Moon, the stars and airglow, as well as on weather and atmospheric conditions (figures 1*e* and 2*a*). The horizontal illuminance *E_V_* (a photometric variable based on human daytime vision) for all light incident on a plane surface, ranges from daytime peaks exceeding 100 000 lx to 800 lx at sunset and a minimum of about 1 mlx during moonless nights, all for clear skies [27]. Illuminance of moonlight varies with Moon position and phase, a maximum of 0.3 lx being reached with a full Moon at zenith [28]. Clouds attenuate light such that illuminance can drop to levels as low as 0.1 mlx in overcast nights [29]. Thus, the total diel range of illuminance from natural light impinging on the Earth's surface spans nine orders of magnitude (figure 1*e*).

### (b) Light pollution

Light pollution was first recognized by astronomers drawing attention to the brightening of the night sky by ALAN. In ecology, light pollution refers to ALAN showing characteristics (irradiance or illuminance, spectral composition, polarization) that deviate from those of the natural nocturnal light regime and exert negative ecological consequences [30]. There are two fundamentally different forms. Direct light pollution is caused by radiation that directly impinges on surfaces, originating from public or, to some extent, private lighting such as street lights, illumination in buildings, vehicles, illuminated advertisements or architectural lighting [31] (figure 1*c*). Levels of up to 20 lx have been measured at the surface of inland waters [22], 70 times higher than the maximum irradiance of moonlight [28]. Indirect light pollution, primarily skyglow (figure 1*d*), which is readily recognized as a light dome above cities, is caused by light radiating horizontally or upwards into the atmosphere, where it is diverted back to Earth, mainly as a result of scattering. Irradiance and the spatial extent of skyglow vary greatly with atmospheric and weather conditions. Clouds in particular amplify skyglow, so that illuminance levels above 1 lx can be reached in urban areas [6]. This is brighter than the brightest moonlight and contrasts with regions unaffected by ALAN, where overcast night skies are much darker than clear skies [32].

The spectrum of ALAN caused by outdoor lighting is currently changing. In parts of the world, it continues to be dominated by gas-discharge lamps such as sodium or mercury vapour lamps. However, the light sources likely to dominate at night in the future are LEDs, with current technology emitting light primarily in the blue range at about 450 nm with a secondary broadband emission at about 600 nm while the ratio of the two peaks can be tuned (red dashed vs. red solid line in figure 2*a*).

### (c) Light attenuation in water

Light is exponentially attenuated in water by wavelength-specific absorption and scattering [33] (electronic supplementary material, S1). Pure water absorbs light weakly at short wavelengths (blue) and strongly at long wavelengths (red). Rayleigh scattering is strongest for short wavelengths and thus results in a blue appearance of clear water bodies. In addition, many dissolved and particulate substances are optically active, including coloured dissolved organic matter (cDOM) such as humic substances, phytoplankton and other suspended particles, which all greatly increase light attenuation. cDOM strongly absorbs light at short wavelengths within the visible range, whereas chlorophyll and other pigments in phytoplankton absorb light across the entire visible spectrum, although not to the same extent [34]. As a result, backscattered light usually dominates in the green region of the spectrum when algal abundances are high, so that many inland waters appear green, whereas waters with high concentrations of cDOM are brown. According to Jerlov's optical classification of marine waters, coastal water type 7C can show brownish, greenish or other colours [24] (figure 2*b*). Conversely, unproductive clear ocean waters lacking optically active constituents (Jerlov water type I) appear blue. The light-changing properties of water and water constituents also affect artificial light, as shown, for example, for light emitted by 5000 K LEDs and high-pressure sodium (HPS) lamps (25 lx) that passes through a 50 m column of clear ocean water or 5 m of turbid coastal or inland water, respectively (figure 2*c,d*). While the blue peak of the LED emission spectrum dominates in clear ocean water where the HPS emission is strongly attenuated, the situation is reversed in turbid waters, where the HPS emission is less attenuated than the blue light emitted by LEDs.

### (d) Refraction, reflection and Snell's window

**Figure 3.**
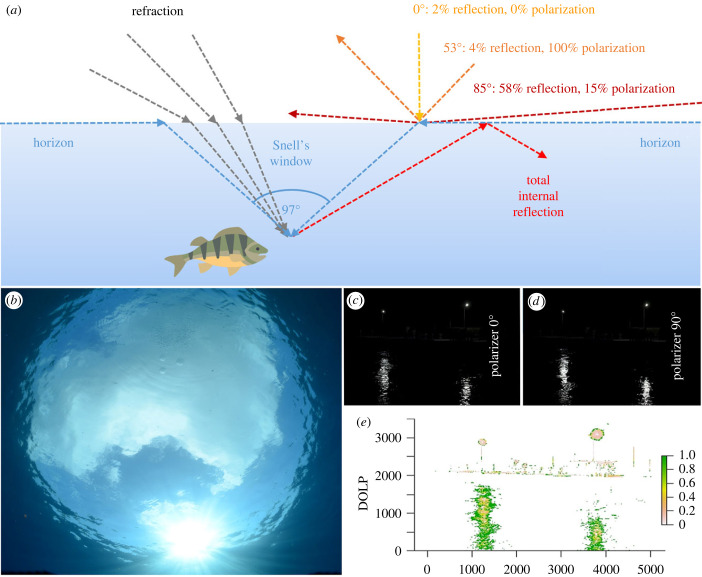
(*a*) Light refraction, reflection and polarization at the air–water interface: unpolarized light impinging perpendicularly on a water surface (incident angle *α* = 0°) remains unpolarized (yellow arrow); the proportion of reflected light incident at *α* = 53°, which is called the Brewster angle, will become fully polarized (orange arrow); and at *α* = 85° (dark red arrow) light will be partially polarized; light rays propagating upwards at an angle greater than 97° are subject to total internal reflection (bright red arrows) and refraction (grey arrows), with the resulting Snell's window (blue arrows). (*b*) Photograph of Snell's window (photo: David K. Lynch and Simon Higton, NASA's ‘Earth Science Picture of the Day’, https://epod.usra.edu/blog/2014/06/snells-window.html). (*c–e*) Illustration and quantification of ALAN polarized at a water surface, with (*c*) showing horizontal and (*d*) vertical polarization effects of LED street lights reflected at the water surface of a river, and (*e*) the degree of linear polarization (DOLP) [35] of the reflected polarized light indicated by colour coding (see electronic supplementary material, S1, for calculations). Fish icon made with Freepik (www.flaticon.com). (Online version in colour.)

When light reaches the water surface, it will be partially reflected and the remaining fraction will penetrate into the water, where it is refracted following Snell's Law (figure 3*a*; electronic supplementary material, S1). For water, refraction leads to an effect called Snell's window, where light that is incident near the horizon (blue arrows in figure 3*a*) creates a 97° wide window (in contrast to the full 180° above the water) with the horizon on land appearing above (figure 3*b*). For light rays propagating upwards in the water outside the window defined by this angle, total internal reflection will occur (bright red arrows in figure 3*a*). Snell's window has implications for fish vision and camouflage of on-shore predators [36,37].

### (e) Light polarization

Most natural and artificial light sources emit unpolarized light, which becomes polarized by scattering in water or the atmosphere, or when being reflected at surfaces such as the air–water interface [38] (figure 3*a*). Polarized light can be viewed as a transversal wave, with the electromagnetic field oscillating perpendicularly to the direction of propagation [35]. If the oscillation is restricted to a single plane, the light is called linearly polarized. If the oscillation occurs equally in all directions and the phase is random, the light is called unpolarized. Where two linearly polarized light fields interfere, the resulting oscillation can rotate during light propagation. This creates elliptically polarized light when the original light fields have different amplitudes or when the phase shift deviates from *π*/2; alternatively, interference of two light fields with equal amplitudes and a phase difference of *π*/2 leads to the special case of circularly polarized light [35]. Since light is polarized when hitting a water surface, with the incident angle determining the magnitude of the effect, rough water surfaces caused by wind-induced ripples and waves affect light polarization by altering the effective incidence angle of light into the water [39], in addition to changing light reflection and penetration into the water body [38]. This can also blur Snell's window.

## 3. The ecological significance of light

### (a) The dual role of light perceived by organisms

Light is of fundamental importance to plants, animals and microorganisms, serving as a source of both energy and information [40]. The single most important process in the biosphere driven by light is photosynthesis, and the importance of light for animal vision is also evident. In addition, however, light determines biological rhythms that are largely driven by changes of diel, lunar and seasonal cycles [41]. Therefore, almost all organisms have genetically determined internal clocks that are tuned by periodic light changes to balance body functions and behaviour according to the time of day, Moon phase or year. Most important are circadian rhythms, which play a central role to ensure homeostasis of organisms, including the repair and recovery of physiological functions during rest or dormancy periods. To synchronize physiological processes, pacemakers conduct the rhythms of cells and organs with their outside environment. A particularly powerful trigger (zeitgeber) is the daily change of light and dark, which in many animals controls the synchronization of the sleep–wake cycle, time course of body temperature and heartbeat, hormonal balance and more [42].

### (b) Underwater light perception

All light-driven biological processes start with light detection, which in water depends on the characteristics of the light source, the optical properties of the water, and the photosensory characteristics of the organisms. Vision under dim light, which is particularly important in deep, stained and turbid inland waters, is facilitated by extended periods of photon capture, by large apertures *A* to maximize overall photon capture, and by short focal lengths *f* of the eye (precisely, large numerical apertures or small *F*-numbers *f*/*A*) to increase the photon density in the image points on the retina [43]. Adaptation to dim light can be effective, allowing even colour vision at light levels equivalent to moonlight (e.g. at 0.02 cd m^−2^ in a freshwater prawn) [44].

Photosensory systems are nearly ubiquitous across the animal and plant kingdom and also occur in microorganisms. Accordingly, photosensory systems and their response to light are highly diverse, ranging from single photoreceptor cells to complex image-forming camera-type eyes, which can capture spatial information and facilitate colour-guided behaviour as well as polarization patterns invisible to humans [11,45]. Blue-shifted light sensitivity, in particular, has been hypothesized to be a conserved trait in all vertebrates in response to the blue-light-rich ocean environment [46]. However, sensitivities to different wavelengths vary among species and habitats where species predominantly occur. Thus, fishes inhabiting well-lit marine waters are often more sensitive to blue light than species inhabiting inland and coastal waters stained by humic substances, such as Eurasian perch (*Perca fluviatilis*) and roach (*Rutilus rutilus*), which are also sensitive to light at longer wavelengths [47–49]. Spectral sensitivities can even change during life-history [50,51] or when preferred habitats of species shift to greater water depths in response to climate-change-induced warming of shallow-water habitats [52]. In particular, aquatic insects and anurans with terrestrial adult stages but aquatic larvae, nymphs or tadpoles experience dramatic changes in light conditions (irradiance, spectral distribution, polarization) during their life-history, requiring adaptations of light perception capacities. Mayfly nymphs of *Ephoron virgo*, for example, are mainly green-sensitive, whereas adults are primarily UV-sensitive [53]. Such changes in visual capacities can occur at levels ranging from corneal and lens structure to neural organization [54,55]. Overall, the examples illustrating variation at multiple levels suggest a strong selection pressure on photosensory capacities.

Organisms perceive visual information in various ways by using suitable photosensitive receptors. This includes receptors capturing UV and near-IR light [11] as well as light polarization [45], all of which are outside of human perception abilities. Most nocturnal aquatic insects, including many that spend most of their life cycle in water (e.g. mayfly, caddisfly and mosquito larvae, as well as many beetles and water bugs), have efficient sensors to detect light at low levels, enabling them even to use starlight for orientation. Many species can also perceive polarized light and use this ability to locate suitable habitats and oviposition sites by orientating along distinct polarization signatures created by reflections on water surfaces [56]. The polarization sensitivity of flying aquatic insects is usually in the UV or blue spectral range, since the degree of polarization of light reflected from natural water surfaces is highest at short wavelengths [57]. Moreover, while in humans and other mammals multiple photoreceptors are concentrated in the retina (including melanopsin), fishes and other vertebrates possess additional, anatomically diverse extraretinal photoreceptors such as the pineal organ, parapineal organ and deep-brain photoreceptors [12].

### (c) Light as a zeitgeber

Natural cycles of light and darkness structure ecosystems along temporal dimensions. The resulting strong selection pressure has led to divergent adaptations of the inner clock of species, giving rise to nocturnal (night-active), crepuscular (active mostly during twilight) and diurnal (day-active) species. About 30% of all known vertebrates and more than 60% of invertebrates are nocturnal [10], and have commonly adapted to the night niche by acquiring highly developed senses, including specially adapted eyesight. Times of foraging or rest as well as the search for mating partners and reproduction are also synchronized with the hours of daylight, darkness or twilight, or with different periods of light and darkness in more complex ways [58]. Such physiological and behavioural species traits controlled by light–dark cycles also affect ecological interactions. Repercussions for the structure of communities and ecosystem processes ensue [9].

One striking example relates to perhaps the largest synchronized movements of biomass regulated by the diel light–dark cycle globally: the diel vertical migrations (DVMs) of zooplankton and fish [59]. Many zooplankton species reside at low light levels in deep waters during the day to minimize predation risk from visually hunting planktivorous fish, moving to surface water layers only at night to feed on phytoplankton and microzooplankton [59]. The benefits of seeking a daytime refuge in deep waters can be gauged from the fact that the strategy has been successful despite the energetic costs of the movement, deprivation from high-quality food during the day, and reduced growth rates at the low temperatures in deep water. Similar to zooplankton, planktivorous fishes in lakes such as whitefish (*Coregonus* spp.) seek refuge under dim light in deep water during the day to minimize predation risk by piscivorous fish and birds, rising to shallower water layers only at night [60]. They thus follow the DVM of their zooplankton prey, but feed much less efficiently than under bright daylight near the surface [61]. The remarkable sensitivity of species to diel changes in light levels is very well illustrated by the circalunar patterns in zooplankton demography, community composition, and consumption by fish in an equatorial reservoir [62]. The low light levels of full Moon are sufficient for fishes in this lake to feed efficiently enough on zooplankton to strongly reduce population densities (Moon trap), which then regularly recover during darker nights between phases of full Moon.

Seasonal (circannual) rhythms are also synchronized by light, especially by daylight length. They are particularly important for reproduction. This includes the spawning migration of many fishes [63], the search for mating partners, spawning, and the development of fry, which are typically cued to periods when food supply is sufficient for offspring survival. Photoperiod can also trigger seasonal habitat shifts to access resources or find suitable conditions for reproduction, in addition to prompting (early) preparations to cope with challenging temperature, food and light conditions during winter. This can involve physiological, morphological and behavioural responses such as migration or hibernation at reduced metabolic activities [64], or the early seasonal accumulation of energy reserves. Accordingly, fish replenish their energy stores before winter, aquatic insects their lipid reserves before emergence, and generally time their life-history stages (eggs, larvae, pupae, adults) to avoid periods of droughts, floods or freezing, and benefit from favourable temperatures and food supplies [64–66].

## 4. Ecological consequences of light pollution

### (a) Artificial light as a challenge to life

**Figure 4.**
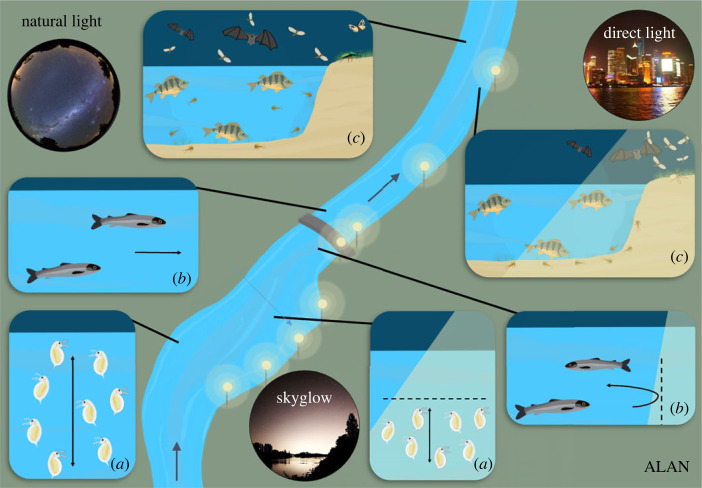
Examples of ecological consequences of artificial light at night (ALAN) along a river–lake continuum, showing interference (*a*) with zooplankton diel vertical migration [15], (*b*) longitudinal migration of fish [98] and (*c*) predator–prey interactions [68,69], including insect drift [70] and effects across the land–water interface [71,72]. The left side of the river illustrates the situation under naturally dark skies, and the right side highlights the impacts of ALAN. Arrows show the direction of river flow. (Online version in colour.)

The consequences of ALAN, whether from direct light sources or skyglow, can be important at all levels of biodiversity, from individuals to ecosystems [7,9]. Interference with biological processes may be due to unnatural light levels, spectral composition, polarization patterns or the timing of illumination, and can lead to the decoupling of biological rhythms of individual organisms and whole ecosystems [40]. At the base of the impacts are changes in physiological processes, body functions and behaviour, which propagate to manifold intra- and interspecific interactions and affect population dynamics, fitness and evolutionary trajectories, food-web relationships, biodiversity patterns and ecosystem processes (figure 4). [13,73]. The underlying mechanisms can be direct, indirect or a combination of both. In addition, ALAN can increase susceptibility to other stress factors such as global warming or water pollution [9].

### (b) Impacts on vertebrate physiology

Artificial lighting clearly alters the physiology of many aquatic organisms (vertebrates, invertebrates, vascular plants, algae, fungi, bacteria). This includes the hormonal balance and various physiological activities cued to diel (e.g. digestion, repair and recovery of physiological functions) and seasonal (e.g. reproduction) rhythms. Disruptions of the normal circadian and circannual timing can hence evoke a multitude of downstream effects, potentially reorganizing the entire physiological state of an organism [74]. Best known is the suppression of nocturnal melatonin production in vertebrates, resulting in a cascade of physiological responses [12]. Some fish species such as Eurasian perch and roach respond by suppressing melatonin release at illuminance levels of white light as low as 0.01 and 1 lx, respectively [14,75]. Furthermore, the melatonin signalling pathway is slightly affected at low levels of ALAN (3 lx) in tadpoles of agile frogs (*Rana dalmatina*) and common toads (*Bufo bufo*) [76].

Similar responses to red, green and blue light suggest that spectral composition plays a marginal role in Eurasian perch and roach [75,77], contrasting with responses of many terrestrial mammals and adult insects, which are particularly sensitive to light at short wavelengths [12,78,79]. A plausible reason for this striking deviance is the wide range of colours encountered by perch and roach in inland waters differing widely in water quality (figure 2*b*).

A variety of other mechanisms could alter aquatic vertebrate physiology in response to light exposure. This prominently includes the control of sexual maturation by seasonal changes in photoperiod. Specifically, gonad development of many fishes in temperate climates is triggered by decreasing daylengths in autumn. If this signal is masked by artificial light during such a photo-labile period, the cue for gonad maturation is lost, as demonstrated for both perch and roach [80]. The fitness consequences could be severe.

The blood concentrations of glucocorticoid hormones such as cortisol are a frequently measured indicator of stress in vertebrates, affecting growth, osmoregulation, immune function and energy metabolism, as well as the transition between resting and waking [23]. Cortisol blood levels proved insensitive to artificial nightlight in several fish species such as perch [81], mosquitofish (*Gambusia affinis*) [82] and juvenile bonefish (*Albula vulpes*) [83]. This outcome suggests that fishes may not always perceive light at night as stress, although nightlight affects the melatonin level and reproductive physiology in fish [75,77]. This means, however, that some species may miss critical information to assess risks associated with ALAN, which could prevent them from avoiding illuminated habitats with potentially detrimental consequences for these species. Tadpoles of Rio Grande leopard frogs (*Rana berlandieri*) and Gulf Coast toads (*Bufo valliceps*) showed a different stress response, with corticosterone levels being increased [84], whereas corticosterone levels of adult cane toads (*Rhinella marina*) were lowered [85].

### (c) Behavioural responses of aquatic insects

One of the most striking effects of ALAN is the attraction of nocturnal flying insects to light sources, where they become easy prey for terrestrial predators or die of exhaustion while circling the light source. Aquatic flying insects appear to be particularly vulnerable [71,86]. Even when this ‘vacuum cleaner effect’ [87] is not immediately fatal, negative consequences will ensue, because reduced search times for food and partners have fitness implications. This is concerning because freshwater insects comprise nearly 100 000 described species, accounting for 60% of the known animal species richness in freshwaters [88]. One reason for the particular vulnerability could be that many flying aquatic insects are guided by linearly polarized light reflected at water surfaces [45], which superimpose effects caused by ALAN at high levels. Such effects have been documented at an illuminated river bridge [89], where flying nocturnal mayflies *Ephoron virgo* were attracted by street lamps emitting unpolarized light while also being led astray by polarized light reflected from the asphalt on the bridge, which was apparently perceived as a sheet of water. Thus, both direct and indirect light pollution interacted in luring large numbers of mass-emerging insects towards an unsuitable oviposition site.

Light sources emitting short-wavelength blue and UV light are especially effective at attracting flying insects [78,79]. However, the importance of spectral composition is not always evident for aquatic species. For example, adult mayflies show positive phototaxis at short wavelengths [90], whereas non-biting midges appear to be rather indifferent [91,92]. Furthermore, while strong positive phototaxis is prevalent in aquatic life stages, similar to flying stages, a preference for short wavelengths is not apparent. This is exemplified by mayfly nymphs, which are not responsive to blue light, and by larvae of non-biting midges as well as water bugs, which respond to green and yellow instead of blue light. These varied responses conceivably reflect adaptations to wavelength-specific light attenuation in different freshwaters [93].

Nocturnal drift is a common behaviour of insects in running waters to avoid predation by fish, reduce competition and find favourable habitat conditions. Several aquatic insect species drift only at light levels below 0.001 lx (measured in lab conditions) [94] or 1 and 2 lx (field conditions) [95], largely independent of wavelength. In another field experiment, drift densities of aquatic invertebrates (mainly baetid mayflies) were reduced by 50% when exposed to artificial light (HPS lamps) at 0.5 lx at the water surface [70]. These results suggest that even low levels of light pollution affect the drift behaviour of aquatic insects (figure 4*c*).

### (d) Light as a movement barrier in water bodies

A potentially important consequence of direct light sources near freshwaters is barrier effects for animal movement. Critical structures include illuminated overpasses and other crossing structures such as bridges and weirs [22]. Some salmonids and eels occasionally interrupt their migration at such lit structures [96–99], suggesting that ALAN can increase landscape resistance in river networks. Such effects are not limited to fish. The lighting of crossing structures and waterways also has adverse consequences for bats that use the linear water courses as flyways and feeding habitats [100], and female mayflies are attracted by bridge lights during upstream compensatory flights [90]. Finally, common toads (*B. bufo*) avoid road sections illuminated with white or green but not red light during annual spring migrations [101]. Thus, light barrier effects on migratory freshwater fauna appear to be common as a consequence of both attraction to and avoidance of ALAN. The required extra time and energy expenditure may threaten synchronous reproduction and reproductive success, again with significant fitness consequences [102].

### (e) Altered predator–prey relationships within and beyond freshwater ecosystems

Predators have evolved sophisticated techniques over millions of years to detect and capture prey, while prey species constantly improve defence strategies. Artificial light at night can shift the balance in this arms race (figure 4*c*). A predator avoidance strategy affected by ALAN is the synchronous night-time dispersal of migratory fishes. Small predatory sculpins (*Cottus* sp.) exploit increased densities of sockeye fry (*Oncorhynchus nerka*) attracted by artificial light during night-time dispersal, independent of the light spectrum [68,103]. Likewise, predation risk for Chinook (*Oncorhynchus tshawytscha*) smolts increased 3–5 h after sunset as light levels increased from 0 to 70 lx at the water surface [104]. When night-time illuminance was low (2 lx), perch (*P. fluviatilis*) consumed significantly more gammarids than on dark nights [69]. Trinidadian guppies, *Poecilia reticulata*, are a case in point in that, beyond affecting nocturnal behaviour, ALAN can affect diurnal behaviour associated with risk taking. When exposed to low light at night (0.5 lx, see also §4g), these fish emerged from a daytime refuge faster than fish kept in natural darkness at night [105]. ALAN thus has the potential to alter predator–prey interactions and to influence species abundances and community structure as a result (figure 4*c*).

Various terrestrial predators, including spiders and bats, benefit greatly from high densities of aquatic insects attracted and disoriented by artificial light sources. The nocturnal orb-web spider (*Larinioides sclopetarius*) typically builds webs at illuminated bridges, where it catches emerging aquatic insects, especially chironomids [106]. Similarly, nocturnal ground-dwelling spiders (*Pachygnatha clercki*, *Trochosa* sp.) and harvestmen have extended their foraging activities into the day when exposed to street lighting, which has been interpreted as a response to benefit from the large numbers of exhausted insects, especially of aquatic species, dropped dead beneath street lamps [71,107]. Finally, artificial light can also influence the insect and bat fauna along rivers. For example, chironomids, ceratopogonids and the activity of Kuhl's pygmy bat (*Pipistrellus kuhliiat*) increased near a light source, whereas the activity of Daubenton's bat (*Myotis daubentonii*) decreased [108]. These responses suggest that altered predator and prey behaviours triggered by street lights can influence food-web relationships and fluxes of food resources across aquatic–terrestrial ecosystem boundaries (figure 4*c*), resulting in altered community structure and dynamics [71,72].

### (f) Novel communities

An important point to note is the substantial variation to be expected in responses to ALAN both among and within taxonomic groups, which could change species distribution patterns and create novel communities with no historical analogue, resulting from the loss of previously established and arrival of new species. An example are cane toads introduced to and highly invasive in various islands throughout Oceania and the Caribbean, as well as Australia, which benefit from insects attracted by street lights, especially in areas with low ambient light pollution [109]. The ‘vacuum cleaner effect’ referring to such aggregation of individuals in artificially illuminated areas [87] reduces abundances of the attracted species in the darker surroundings. Conversely, densities of species repelled by light would increase in dark areas adjacent to illuminated sites [86]. ALAN near freshwaters could even provide a night-time energy source for microbial phototrophs (cyanobacteria, diatoms, green algae) capable of growing at low light levels. This, too, could induce community shifts towards microbial taxa that benefit from nocturnal light [17,67]. Short-wave light appears to be particularly prone to triggering such effects on periphyton communities developing near lit water surfaces (less than 15 cm) [110], where spectral attenuation of short wavelengths can be expected not to be pronounced.

### (g) Skyglow as a driver

Indirect light pollution by skyglow could be particularly important for lakes, especially large ones, where any radiation emitted from light sources on the shoreline tends to be relatively far away and impinge on the water surface at a shallow angle, so that most of the incident direct light from nearby light sources is reflected (figure 3*a*). Skyglow, by contrast, hits the water surface from practically all angles above the horizon, resulting in a much larger fraction of the incident light penetrating the water. If organisms respond to the low irradiance levels that are characteristic of skyglow, the consequences for biodiversity and ecosystems could nevertheless be far-reaching [111]. A striking example relates to the diel vertical migration of zooplankton, which was disrupted under urban skyglow and re-established by experimental dimming in a lake enclosure experiment [15] (figures 1*f* and 4*a*). The zooplankton movement suppressed by skyglow would be expected not only to have repercussions for fish feeding on their zooplankton prey. Phytoplankton could be affected as well by benefiting from reduced grazing pressure under skyglow. This would occur both when zooplankton abides in the deep-water refuge also at night, and when zooplankton is eaten by planktivorous fish that feed more efficiently under improved night-time light conditions in surface water layers. Knock-on effects on species interactions within phytoplankton communities and with other community members (e.g. bacteria) are likely, although the consequences on plankton food webs and lake productivity currently remain unclear. However, the extraordinary sensitivity as well as physiological and behavioural responses of fish to skyglow light levels [14,105] imply that altered fish behaviour could have implications for both the fishes themselves and any species interacting with them.

### (h) Evolutionary consequences

Species traits affected by artificial light can undergo evolutionary change. Some species already have adapted, whereas others may fail [73]. The new bright-light situation at night is expected to favour insensitive over photosensitive genotypes, especially in urban water bodies, where the selection pressure is strongest [111,112]. However, if such adaptations occur, they would reduce genetic diversity, because genotypes poorly adapted to nocturnal light would decline. Furthermore, ALAN can indirectly affect evolutionary trajectories by influencing important selection factors. For example, female túngara frogs (*Engystomops pustulosusis*) are less selective in their choice of males at elevated light levels, possibly to reduce predation risk by visual predators during mating [113]. Another example is caddisfly species where males and females are differentially attracted to artificial light [114], which can reduce effective population size and genetic diversity [115], both important population features facilitating adaptation to new conditions. Thus, light pollution can reduce the evolutionary potential of populations to cope with changing environments, with possible consequences for the structure of communities and ecosystem processes.

### (i) Effects on ecosystem processes

ALAN at levels approaching extreme skyglow (6.6 lx) has been found to have photophysiological effects on cultures of cyanobacteria [16], but proved too low to affect net photosynthesis or growth of the cultures. Similarly, somewhat higher illumination levels (20 lx) were insufficient to enable effective photosynthesis of periphyton in experimental flumes placed along an alpine stream and an agricultural drainage ditch [110,116]. On the contrary, periphyton biomass in the flumes declined at elevated light levels. A possible explanation for this unexpected outcome is that the energetic costs for keeping the photosynthetic machinery running at night exceeded the gains that photosynthesis could achieve at the low light level [17]. This would suggest that light pollution must exceed a threshold well above skyglow to notably affect primary production, the single most important ecosystem process in the biosphere. Brighter nocturnal light (71 lx), however, equivalent to high levels of direct street lighting (figure 1*e*), facilitated photosynthesis [17] and shifted net ecosystem production at night from negative to positive. This shift is a remarkable phenomenon, unprecedented in Earth's history.

Heterotrophic ecosystem processes can also be affected by ALAN, although information on such effects is currently scarce. Although CO_2_ and CH_4_ emissions were not systematically associated with light pollution across a large number of inland waters [67], sediment respiration in a ditch was reduced under the influence of night-time illumination [17]. Furthermore, rates of microbial leaf-litter decomposition increased in aquatic microcosms exposed to ALAN, accompanied by changes in potential activities of extracellular enzymes. However, the stimulating effect on decomposition was not consistent across experiments [117,118] and the underlying mechanism remains obscure at present. Finally, ALAN increased ingestion rates of litter-consuming detritivores, apparently owing to compensatory feeding by shredders and grazers such as gammarids [119] and snails [120]. These preliminary findings point to the intricacy of assessing ALAN effects on heterotrophic ecosystem processes, especially if taxonomically and functionally diverse decomposer communities are involved.

### (j) Stressor interactions

The potential for ALAN to produce interactive effects with other stressors is large, particularly in urbanized areas where light pollution is greatest and many other stressors are present. This can make it challenging to disentangle the specific role of artificial light in causing adverse effects on biodiversity and ecosystem functioning. Heat and noise pollution commonly occur in concert with light pollution in urban settings, and so does water pollution by nutrients, heavy metals and synthetic chemicals. All of these stressors can affect the physiology, behaviour, abundance, spatio-temporal distribution and evolutionary trajectories of species, as well as species interactions and ecosystem processes. It is essentially unknown to what extent ALAN and those stressors interact and whether the outcomes are additive, synergistic or antagonistic [9]. Nevertheless, two examples illustrate the complexity of the type of interactions to be expected. First, the abundance of túngara frogs (*Engystomops pustulosus*) was not correlated with light, noise or warming [121]. However, frog-biting midges (*Corethrella* spp.) parasitizing the frogs were sensitive to both light and noise pollution; light reduced midge abundance at low noise levels but had no effect at high noise levels, where midges were absent regardless of light exposure. Furthermore, ALAN alleviated the adverse effects of both silver nanoparticles and lead on litter decomposition and the composition of the associated fungal communities [117,118]. These first findings underline the need to consider ecological impacts of ALAN in the context of multiple stressors.

## 5. Towards environmentally friendly electric lighting

Alleviating light pollution requires innovative concepts that include the protection of biodiversity and ecosystems as an explicit objective. With some progress in developing environmentally friendly lighting concepts, the importance of reducing light pollution has indeed been increasingly recognized [9]. However, despite evidence of effective, readily available and even inexpensive mitigation strategies (e.g. light orientation, shielding, scaling of light levels to the intended use, spectral tuning [1,122,123]), little attention has been given to measures that specifically address light pollution of inland waters and surrounding land [21,22].

Adapting lighting levels to specific jeopardies is a first important principle, which involves both a temporal and spatial component. Illumination can be switched off or dimmed at during photo-labile periods concerning reproduction or animal movements, such as seasonal fish migrations or synchronized mass emergence of mayfly species. Spatially, a similarly cautious strategy is to avoid illumination at sensitive locations, including bridges or lakefront and riverside streets and promenades, and never to illuminate water surfaces directly. Second, in addition to reducing luminous flux, which can be finely adjusted with LED luminaires, radiation geometry should direct light effectively and exclusively to those areas that are to be lit. The goal is to prevent light from impinging on water surfaces or other sensitive habitats, and from being emitted, directly or indirectly, either upward or horizontally to produce skyglow [124]. Thirdly, measures to safeguard freshwater biodiversity must encompass adjacent terrestrial ecosystems, because most aquatic insects and various aquatic vertebrate species complete part of their life cycle on land. Finally, differences in spectral sensitivities of all species and life stages present must be considered when planning lighting installations, not only near inland waters. An important recent recognition, however, is that mitigation by spectral tuning, as applied to insect flight or terrestrial mammals and consisting of reducing specific, generally short-wave light emissions, is unlikely to be effective for the protection of inland waters, because many aquatic animals exploit the entire visible spectrum. Consequently, protective measures such as improving luminance distribution or reducing light levels and duration are likely to be much more effective at minimizing negative consequences for freshwater biodiversity and ecosystems.

## 6. Conclusion

The rapid increase in emissions from nocturnal artificial lighting continues unabated worldwide [3]. This alters night-time light levels along with the spectral composition, frequency and duration of light impinging on water surfaces, both by direct exposure in urban settings and indirectly through skyglow in rural and more remote areas. The ecological consequences extend beyond terrestrial ecosystems where most light pollution originates, affecting lakes, rivers and other surface waters alike [21,22]. Moreover, many aquatic insects and other taxa (e.g. Amphibia) complete part of their life cycle on land and thus face a double risk from artificial light in aquatic and terrestrial environments. The primary effects of light at night on the physiology and behaviour of species can propagate to all levels of ecological organization, from individuals to ecosystems [9]. Furthermore, habitat use can be compromised not only for nocturnal but also for diurnal species—directly as a result of altered diel rhythms and indirectly by altered species interactions. All of these effects can be exacerbated by additional stress factors, including climate warming and water pollution.

Large knowledge gaps remain in understanding the nature of light pollution, the ecological consequences for inland waters, and ways to mitigate them. Many of the pressing issues have recently been summarized [9], underlining the multidisciplinary nature of research on light, light pollution and mitigation of the resulting adverse effects. These issues almost invariably apply to freshwaters as much as to terrestrial ecosystems, which have received most attention to date. It is clear, however, that the properties and propagation of light impinging on water create particular conditions that must be considered when assessing and aiming to curb negative consequences of ALAN for freshwaters.

Unresolved issues range from methodological challenges in standardizing light measurements, particularly for skyglow and polarized light, to impacts on multiple aspects of biodiversity, species interactions, eco-evolutionary dynamics and ecosystem processes, especially in multi-stressor contexts, which are the rule not only in urban environments. Equally important are advances in the design and technical implementation of sustainable lighting that minimizes ecological impacts, as well as an improved understanding of society's responses to technical and legislative measures, whether from a psychological, social or policy perspective [9].

While by no means comprehensive, this list illustrates the wide variety of aspects awaiting exploration and solutions. To single out just one example, much remains to be learned about how aquatic macrophytes respond to ALAN, since assessments of physiological effects on freshwater organisms have almost exclusively focused on animals. Nevertheless, the limited information available to date suggests that at least two genera of submerged plants, *Myriophyllum* and *Potamogeton*, show physiological responses even at light levels as low as 3 lx [125]. Furthermore, there is evidence for effects on plant–herbivore interactions, given that *Ceratophyllum* exposed to ALAN becomes more palatable to a freshwater snail, *Lymnaea stagnalis* [126].

In view of the range of demonstrated and potential impacts of light pollution on freshwaters, it is imperative to develop and apply effective lighting and light protection concepts. From a technical perspective, many measures are easy to implement. Where knowledge gaps exist, which is frequently the case, the precautionary principle must be used [9]. A critical additional prerequisite to alleviating impacts of ALAN is to sensitize citizens and decision makers to the problem of light pollution, building on the recognition that light serves as a key source of information to almost all living beings, profoundly influencing metabolism and behaviour. Embedded in comprehensive policies and strategies [127], these measures will greatly improve the protection of freshwater biodiversity and ecosystems from ALAN without foregoing the benefits of nocturnal lighting for humans.

## Data Availability

This article presents no original data.
